# How Does Quinoa (*Chenopodium quinoa* Willd.) Respond to Phosphorus Fertilization and Irrigation Water Salinity?

**DOI:** 10.3390/plants11020216

**Published:** 2022-01-14

**Authors:** Hamza Bouras, Redouane Choukr-Allah, Younes Amouaouch, Ahmed Bouaziz, Krishna Prasad Devkota, Ayoub El Mouttaqi, Bassou Bouazzama, Abdelaziz Hirich

**Affiliations:** 1 Department of Crop Production, Protection and Biotechnology, Hassan II Institute of Agronomy and Veterinary Medicine, Rabat 10101, Morocco; bourashamza07@gmail.com (H.B.); redouane53@yahoo.fr (R.C.-A.); amouyou@gmail.com (Y.A.); hmadbouaziz@gmail.com (A.B.); 2 Biosaline Agriculture Research Program, African Sustainable Agriculture Research Institute (ASARI), Mohammed VI Polytechnic University (UM6P), Laayoune 70000, Morocco; krishna.devkota@um6p.ma (K.P.D.); Ayoub.elmouttaqi@um6p.ma (A.E.M.); 3 Agricultural Research Regional Center of Tadla, National Institute for Agricultural Research (INRA), Beni Mellal 23020, Morocco; bassoubouazzama@gmail.com

**Keywords:** *Chenopodium quinoa*, salinity, phosphorus, biomass yield, seed yield

## Abstract

Soil salinity is a major problem in arid and semi-arid regions, causing land degradation, desertification, and subsequently, food insecurity. Salt-affected soils and phosphorus (P) deficiency are the common problems in the sub-Sahara, including the Southern region of Morocco. Soil salinity limits plant growth by limiting water availability, causing a nutritional imbalance, and imparting osmotic stress in the plants. The objective of this study was to determine the positive effects of P on growth and productivity and understand the major leaf mineral nutrient content of quinoa (*Chenopodium quinoa* Willd.) cv. “ICBA Q5” irrigated with saline water. A field experiment applying three salinity (Electrical Conductivity, EC) levels of irrigation water (ECw = 5, 12, and 17 dS·m^−1^) and three P fertilizer rates (0, 60, and 70 kg of P_2_O_5_ ha^−1^) were evaluated in a split-plot design with three replications. The experiment was conducted in Foum El Oued, South of Morocco on sandy loam soil during the period of March–July 2020. The results showed that irrigation with saline water significantly reduced the final dry biomass, seed yield, harvest index, and crop water productivity of quinoa; however, P application under saline conditions minimized the effect of salinity and improved the yield. The application of 60 and 70 kg of P_2_O_5_ ha^−1^ increased (*p* < 0.05) the seed yield by 29 and 51% at low salinity (5 dS·m^−1^), by 16 and 2% at medium salinity (12 dS·m^−1^), and by 13 and 8% at high salinity (17 dS·m^−1^), respectively. The leaf Na^+^ and K^+^ content and Na^+^/K^+^ ratio increased with irrigation water salinity. However, the leaf content of Mg, Ca, Zn, and Fe decreased under high salinity. It was also found that increasing P fertilization improved the essential nutrient content and nutrient uptake. Our finding suggests that P application minimizes the adverse effects of high soil salinity and can be adopted as a coping strategy under saline conditions.

## 1. Introduction

Soil salinization is a global problem affecting agricultural productivity, and salt-affected soils have been spread over 100 countries. Of the total cultivated land, 227 Mha is irrigated, and out of those, 20% is salt-affected [1,2]. In addition, out of the nonirrigated cultivated land, an additional 2.5% also suffers from salinity [3]. Soil salinization is expanding at a rate of 1–2 million ha·year^−1^ globally, offsetting a significant portion of crop production and making land unsuitable for cultivation. In dryland regions, as soil evaporation greatly exceeds precipitation, crop production mostly depends on irrigation and is vulnerable to soil salinity [4].

The salt tolerance of a crop species is the ability of the crop to grow and produce a harvestable yield in soils affected by salinity [5,6]. Among the crop species adapted to salt-affected drylands, quinoa (*Chenopodium quinoa* Willd.) is considered an alternative crop with a high potential for maintaining farm productivity [7,8]. It is seen as a crop with a high potential to contribute to food, forage, and nutritional security, especially in marginal environments [9]. Quinoa is a facultative halophyte that is not only adapted to high soil salinity but also tolerant to other abiotic stress such as drought and heat [8], ultraviolet B radiation [10], and low temperature [11]. It grows well with no or limited yield loss with an irrigation water salinity up to 10−20 dS·m^−1^ and is able to survive in soils with a salinity level up to seawater (>40 dS·m^−1^) [12,13] and in soil as high as 51.5 dS·m^−1^ [14,15]. Some genotypes even produce higher yields at an irrigation water salinity of 10–20 dS·m^−1^ than under freshwater irrigation. However, other studies have shown yield penalties with an irrigation water salinity level higher than 8 dS·m^−1^: a 50% yield reduction at 25 dS·m^−1^ and a low survival of plants at an irrigation water salinity of 50 dS·m^−1^ [14,16,17].

Soil salinity affects the performance of plants by affecting several physiological processes. The presence of ions (Na^+^ and Cl^−^) in the growth medium (soil) affects water and nutrient uptake. At a high salinity level, turgor pressure reduces in the leaf, the leaf stomata closes, plant photosynthetic rates decline, and plant growth stops [18]. Salinity stress impacts the growth and yield of quinoa directly by reducing metabolism (declining carbon assimilation, decreasing cell expansion, and increasing stomatal closure) and also indirectly through activating metabolic activities in response to salinity stress [19]. It affects the acquisition of mineral nutrients of glycophytes in two ways: (a) it directly affects nutrient uptake and translocation, such as the absorption and accumulation of phosphorus (P) in certain cultivars; (b) it affects the mineral nutrition balance, acquisition, and translocation of nutrient ions within the plant. The higher concentration of Na^+^ and Cl^−^ ions influence nutrient uptake through competitive interactions or by influencing the ionic selectivity, for example, Na^+^ induces Ca^2+^ and K^+^ deficiencies, and Mg^2+^ deficiencies are induced by Ca^2+^ [20].

To cope with salinity stress, tolerant plant species develop defense mechanisms such as the following: (a) they avoid ion uptake by excluding Na^+^ and Cl^−^ from leaves and relying on organic solutes for osmotic adjustment (ion exclusion); (b) they uptake ions, sequester them into the vacuole, and use them as osmotica (tissue tolerance) [21]. Plants adjust their leaf water potential by accumulating salt ions in the cytoplasm, maintaining cell turgor pressure, and limiting their transpiration rate [22]. Salinity-tolerant plant species reduce their transpiration rate under high salinity [16,23], and this reduction in transpiration cuts back the accumulation rate of Na^+^ and Cl^−^ within the leaves. Munns et al. [5] recorded that most of the plant species are able to exclude 98% of the salt within the root system and rootzone. Similarly, vacuolar compartmentalization of Na^+^ and Cl^−^ ions is another osmotic adjustment mechanism of quinoa to tolerate salinity. In this mechanism, the plant compartmentalizes those ions in the vacuole and produces specific species-compatible solutes such as glycine betaine, proline, inositol, pinitol, sorbitol, and mannitol in the cytoplasm. The sequestration of Na^+^ and Cl^−^ within the vacuole not only protects the cytoplasm against toxicity but also increases the diffusion potential of the cell by adjusting the cytosolic diffusion potential [15]. Likewise, ionic dynamics is another defense mechanism, where the K^+^ concentration (which plays a role in the osmotic regulation) in leaves increases by more than 100% due to prolonged and severe salinity [3]. Under normal conditions, the ratio Na^+^/K^+^ concentration in shoots is between 0.5 and 1 [3]. The regulation of K^+^ homeostasis is an important aspect of salt tolerance, and an optimal K^+^/Na^+^ ratio is considered crucial for tolerance or adaptation to salt stress [24].

Appropriate fertilization management is one of the important strategies for dealing with the negative effects of salinity on plants [25]. Phosphorus, an essential (nonsubstitutable) plant nutrient that is deficient, is a widespread problem, and more than 30% of the world’s arable soil is affected due to P deficiency [26]. The optimal amount of P application enhances agronomic and environmental sustainability and food security by balancing soil nutrient availability, improving crop productivity, and minimizing soil nutrient mining [27]. Unraveling the P response, understanding the mechanism by which the P supply makes other nutrients available, optimizing the P application rate, and understanding its role in physiological processes can provide future directions for improving the agronomic and environmental sustainability of salt-affected drylands [28]. Depending on the severity of salinity stress, adding limiting nutrients can increase plant tolerance to salinity [20]. Increasing the P supply enhances the salt tolerance in sesame by improving the interactive effect of osmotic potential and P concentration on water relations, by improving the supply of mineral nutrition and by improving yield characteristics [29]. Increasing the P supply reduces the absorption of toxic ions such as Na^+^ that helps plants avoid salinity damage [30]. However, under saline soils, P availability is reduced due to a (a) reduced activity of orthophosphate ions (H_2_PO_4_^−^ and HPO_4_^2−^), and the sorption processes; and a (b) lower solubility of Ca–P minerals, which control the concentrations of P in the soil solution [31]. The consumption of P fertilizers has been shown to improve yield and sugar content, as well as stomatal conductance under saline irrigation [32]. Under saline conditions, the application of P fertilizers increases the effectiveness of nitrogen fertilizers in corn [33]. Under saline conditions, P application helps to maintain relatively low levels of Na^+^ and high levels of K^+^ in the immature leaves [34]. This suggests that P fertilization conferred greater cellular tolerance to Na^+^ accumulation in older leaves, possibly improving the compartmentalization of intracellular ions. The synergistic relationship between P and other beneficial elements such as K^+^, Ca^2+^, and Mg^2+^ may stabilize the osmotic effect and increase the salinity tolerance to some extent [35]. The increase in P indirectly increases the absorption of Ca^2+^ and Mn^2+^, which could be the cause of the decrease in sodium absorption by the plant [36]. Increasing the phosphorus level from 3 µM to 60 µM in barley nutrient solution grown under salt stress decreases the sodium content [37]. Thus, the objective of this study was to understand the potential of P fertilization for reducing the salinity effect and to determine the interactive effects of irrigation water salinity and P rate on the biomass, seed yield, and leaves mineral content of quinoa.

## 2. Results

### 2.1. Analysis of Variance of Growth Parameters and Leaf Nutrient Concentration

Table 1 summarizes the results of the ANOVA (analysis of variance) for all investigated parameters as affected by irrigation water salinity and phosphorus rate. Irrigation water salinity significantly affected (*p* < 0.05) all parameters except potassium, while phosphorus application had a significant effect on most of the parameters of growth and leaf nutrient content except leaf N, Ca, Zn, and Fe. The interaction between irrigation water salinity and P rate was significant only for a few parameters such as harvest index, P, K, Ca, and Na (Table 1). The significant interaction effect indicated that the effect of P rate varied across the irrigation water salinity.

### 2.2. Seed and Biomass Yield and Harvest Index

**Figure 1:**
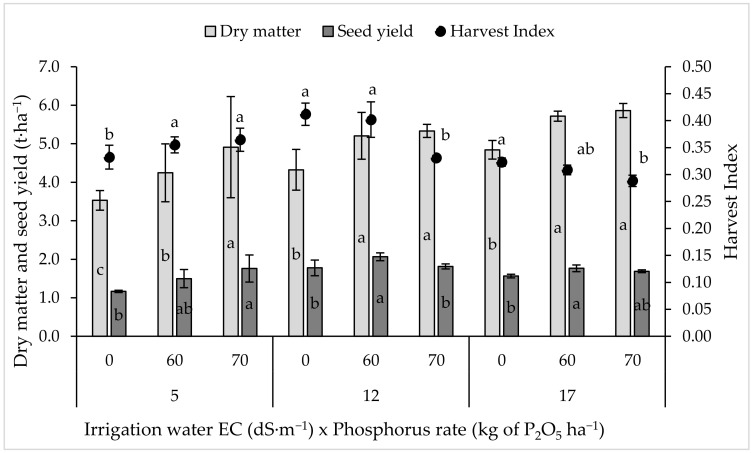
Seed yield, dry matter yield, and harvest index of quinoa as affected by irrigation water salinity and phosphorus rate. Any two values within a column are significantly different (*p* ≤ 0.05) if they have no letter in common. The same letters indicate the statistically homogeneous groups. Error bars indicate the standard deviation.

Irrigation water salinity significantly affected (*p* < 0.05) the final dry biomass, seed yield, and harvest index (HI) (Figure 1). The results showed that the highest dry biomass yield was recorded under the highest irrigation water salinity (EC 17 dS·m^−1^). The gradual increases in the salinity level resulted in significant increases in the biomass production of quinoa, with values of 17 and 29% higher at saline irrigation with EC values of 12 and 17 dS·m^−1^, respectively, compared with the control. The effect of phosphorus fertilization on dry matter production under the tested salinity level was obvious. The application of 60 kg of P_2_O_5_ ha^−1^ increased the dry matter yield by 20, 20, and 18% under low (5 dS·m^−1^), medium (12 dS·m^−1^), and high (17 dS·m^−1^) salinity levels, respectively, compared to the control (0 kg of P_2_O_5_ ha^−1^), while the application of 70 kg of P_2_O_5_ ha^−1^ led to an increase in dry matter by 39, 23, and 21% under low, medium, and high salinity levels, respectively, compared to the control (0 kg of P_2_O_5_ ha^−1^). The obtained results clearly indicated that quinoa better responded to an incremental rate of phosphorus under low salinity compared to medium and high salinity.

Similar to dry biomass, the seed yield of quinoa was significantly increased under medium and high salinity compared to low salinity. Saline irrigation water with EC values up to 12 and 17 dS·m^−1^ significantly increased the average seed yield of quinoa by 27 and 13% compared to the control, respectively. Regarding P rate, it was observed that an application of 60 kg of P_2_O_5_ ha^−1^ increased the dry matter yield by 29, 16, and 13% under low, medium, and high salinity levels, respectively, compared to the control, while the application of 70 kg of P_2_O_5_ ha^−1^ led to increases in dry matter of 51, 2, and 8% under low, medium, and high salinity levels, respectively, compared to the control. In dry matter yield, the effect of P fertilization was more pronounced under low salinity compared to medium and high salinity levels.

The harvest Index (HI) varied considerably with an increasing rate of salinity and P rate. Under low salinity conditions (5 dS·m^−1^), P fertilization significantly improved the HI, while under medium and high salinity, the HI response declined with P rate. The reduction in HI can mainly be explained by the increase in dry matter and relative reduction in seed yield.

### 2.3. Crop and Irrigation Water Productivity

**Figure 2:**
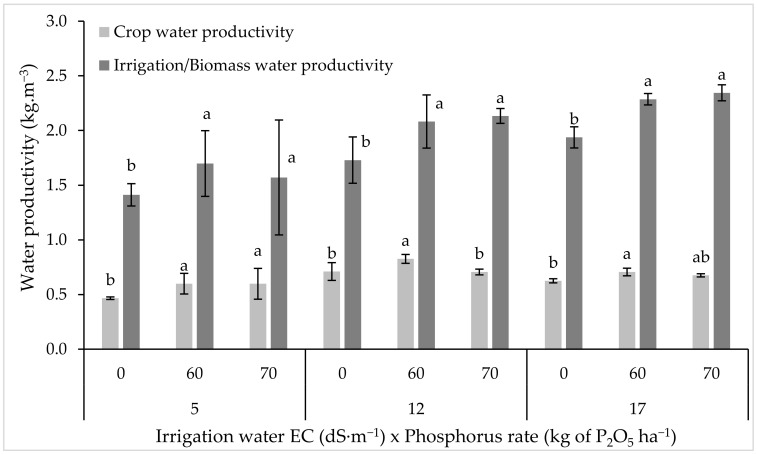
Variation in crop and irrigation water productivity under different phosphorus fertilizer rates and irrigation water salinities. Any two values within a column are significantly different (*p* ≤ 0.05) if they have no letter in common. The same letters indicate the statistically homogeneous groups. Error bars indicate the standard deviation.

Both crop water productivity (CWP) and irrigation/biomass water productivity (IWP) were positively influenced by the salinity of the irrigation water, and both followed the same trend as dry matter and seed yield (Figure 2). Under all irrigation water salinity levels, IWP was positively affected by P fertilization, while CWP responded differently to P fertilization. In fact, under medium and high salinity conditions, CWP slightly decreased in response to the increased P rate.

### 2.4. Leaf Mineral Content

The nutrient content in the leaf was affected by both irrigation water salinity and P application (Table 2). Irrigating with saline water significantly increased the mineral nutrient content in quinoa leaf, where leaf N, P, K, and Na contents increased significantly, while OC and Ca contents decreased (*p* < 0.05) and no effect was observed for Mg, Zn, and Fe. The effect of P fertilizer rate varied from one salinity level to another. For example, under low salinity (5 dS·m^−1^), P application significantly increased K, Zn, Fe, and Na contents in the leaf; however, it significantly reduced P and Ca. On the other hand, OC, N, and Mg contents in the leaf did not respond to increasing P rates. Under medium salinity, the response of leaf nutrient content to an increased P rate changed, where an increase in P application led to increases in P, K, Mg, Ca, and Zn and a decrease in Na content. Under a high salinity level, the application of 60 kg of P_2_O_5_·ha^−1^ led to increases in the leaf P, K, Mg, Ca, and Zn contents. Under high salinity conditions, the highest P rate led to the accumulation of the highest amount of Na. It was also observed that P fertilization had no effect on leaf N and Fe contents under medium and high salinity levels.

### 2.5. Correlation Matrix

**Figure 3:**
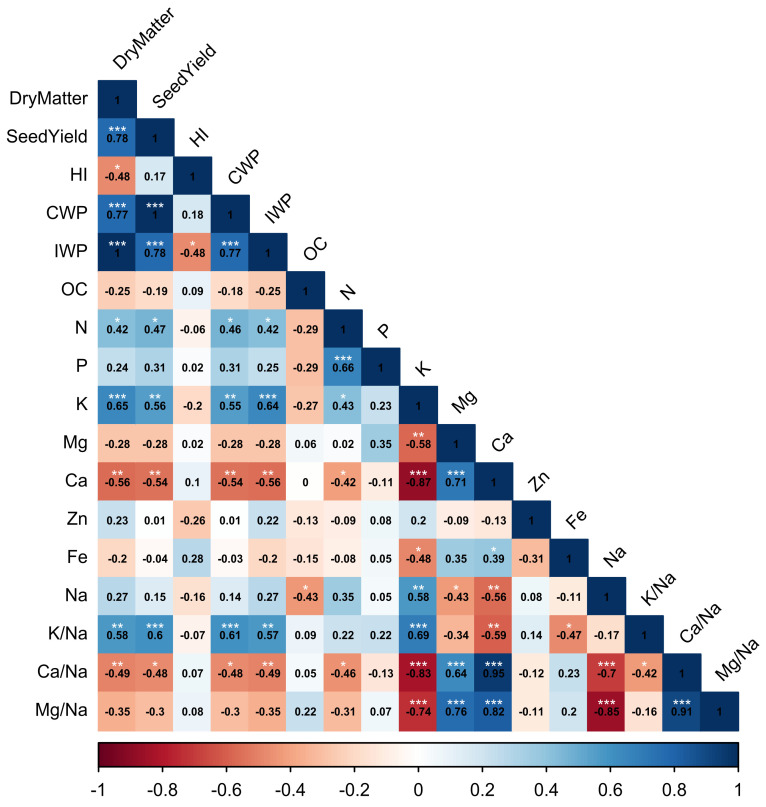
Correlation among seed and dry matter yield, harvest index, crop and irrigation water productivity, and different leaf nutrient contents. *, **, and *** indicate significant differences at *p* < 0.05, 0.01, and 0.001, respectively. The color gradient scale indicates the Pearson coefficient of correlation.

Figure 3 shows the correlation between several measured parameters. There was a significant correlation between productivity parameters (seed and dry matter yield) and several measured parameters such as CWP, IWP, N, K, Ca, K/Na, and Ca/Na. The correlations of those productivity parameters with CWP, IWP, K, and K/Na were positive, while their correlations were negative with Ca and Ca/Na. However, the Na content of the leaf had a significant positive correlation with K, and both of these elements had significant negative correlations with Mg and Ca.

### 2.6. Principal Component Analysis (PCA)

**Figure 4:**
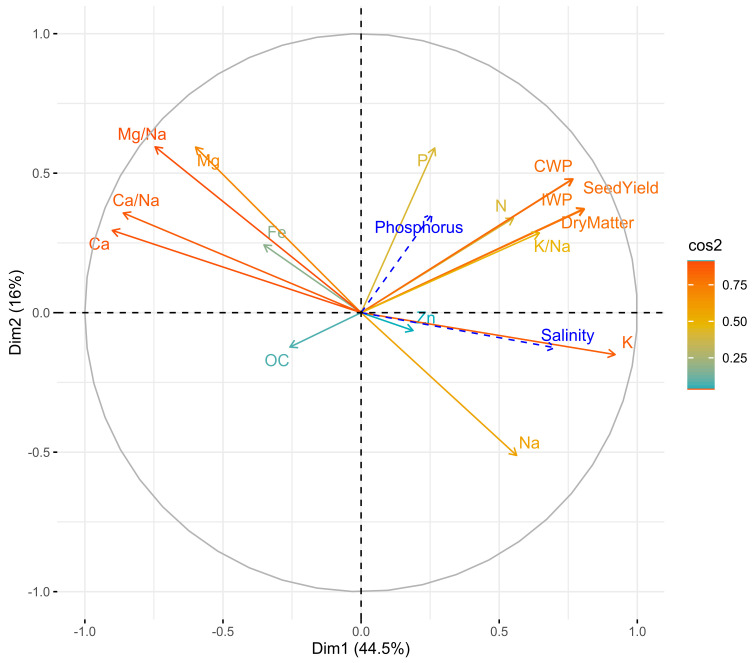
Correlation circle of variables on the first two principal components. Color gradient corresponds to the quality of representation of the variables using the cos^2^ of its coordinates.

Figure 4 shows the quality of representation of the variables by cos^2^. The results of PCA indicated that the first two principal components explained 60.5% of the data variability. PC1 was explained by K, dry matter, IWP, Ca, and Ca/Na. P, Mg, and Mg/Na, despite their moderate correlations, were the main variables that explained the variability present in the PC 2. Leaf Na content did not contribute significantly to explaining the variability present in both axes. The projection showed that salinity was positively correlated with K and negatively with the Ca content of the leaf. Furthermore, applied phosphorus was positively and moderately correlated with leaf P content.

## 3. Discussion

### 3.1. Effect of Salinity Level on Biomass and Seed Yield of Quinoa

Our study clearly showed that saline water significantly affected the growth and productivity parameters of quinoa (Figure 1). The biomass accumulation rate was stable and unaffected by irrigation water salinity up to 17 dS·m^−1^, indicating the great tolerance of quinoa (*Chenopodium quinoa* Willd.) cv. “ICBA Q5” to salinity. In salt-affected drylands, tolerant quinoa species with high dry matter production support livestock production systems [38]. Our results support the earlier finding of Roman et al. [15] who reported that quinoa dry biomass accumulation reduces only at a salinity level of 40 dS·m^−1^ (for 4 weeks). The highest soil salinity at which quinoa is able to survive is 51.5 dS·m^−1^, while at an EC value of 25 dS·m^−1^, the yield can be reduced by 50% [14]. Similar results were obtained by Long [39] who reported that quinoa tolerates fairly high soil salinity. However, Long [39] found reduced growth and yield attributes (plant height, the number of branches and leaves, shoot dry weight, root length, root dry weight, SPAD Chlorophyll Meter, panicle length, the number of branches per panicle, and 1000-seed weight) of quinoa under high soil salinity (>40 dS·m^−1^; 300 mM NaCl) in Vietnam.

A significant decrease in seed yield beyond an EC value of 12 dS·m^−1^, while a significant increase in seed yield between EC 5 and 12 dS·m^−1^ (Figure 1), indicated that quinoa produces a higher seed yield under medium salinity (EC 12 dS·m^−1^) than under low or high salinity levels. Our finding is aligned with the previous results obtained by Hirich et al. [13], who reported that if the salinity of irrigation water exceeds 12 dS·m^−1^, the seed yield begins to decrease. An increment in salinity level reduces the growth and the productivity, HI, and CWP of quinoa; however, the reduction was not severe under high salinity levels (Figure 1). In addition, seed yield seems to be less affected by salinity level than biomass yield. Irrigation with saline water of 20 and 30 dS·m^−1^ reduced grain production by only 24 and 34%, respectively, compared with freshwater (EC 1 dS·m^−1^) [13]. The seed weight of quinoa decreased under a high sodium concentration, and the dry matter reduction (mainly carbohydrates and other carbon-containing molecules) was compensated by an increase in ash content [9]. Seed yield, HI, protein content, 1000-seed weight, and shoot dry matter decreased at high salinity (>20 dS·m^−1^) [40]. Irrigation water salinity (EC 15 dS·m^−1^) significantly decreased the seed yield of quinoa [8], and this reduction was related to the reduction in the number of seeds due to the reduction in the number and size of the inflorescences per plant, which was the main factor contributing to the yield. Similar results in quinoa were also reported by Long [39] in Vietnam where seed yield was reduced under NaCl concentrations from 50, 150, and 300 mM.

### 3.2. Effect of Phosphorus Rate on Biomass and Seed Yield

Quinoa responded positively to an additional supply of phosphorus fertilization at both salinity levels of irrigation water (Figure 1). The negative impact of salt stress on biomass yield was significantly reduced by P application at low-salinity irrigation water (EC 5 dS·m^−1^). The phosphorus supply significantly increased dry matter and seed yield at higher salinity levels. This result is in agreement with the findings of Ghazi and Al-Karaki, 1997 and Khosh et al., 2012 [37,41] in barley. They found that an increment in salinity level significantly reduced the shoot and the root dry weights of barley, while the P supply under saline conditions increased the plant dry weight and subsequently the plant resistance against salt stress. Phosphorus application mitigated the adverse effects of salinity on maize [42,43], mung bean [44], green bean [45], chickpea [46], wheat [47], and sugar beet [32]. Its application increased the yield by increasing the concentration and uptake of essential plant nutrients necessary for plant growth, improving the ratio of Ca/Na and K/Na, and decreasing the concentration of toxic ions (Na^+^ and Cl^−^) [48].

### 3.3. Leaf Mineral Content

The wide variation in ion uptake and ratio across the salinity level and P rates indicated that the quinoa response under salinity varies with P application rate (Table 2). Quinoa plants are able to maintain the uptake of potassium despite high sodium concentrations in the soil after prolonged salt stress (16 weeks after the start of the stress) [3]. When plants are transferred into a medium with a high Na^+^ concentration (salt treatment), the K^+^ concentration on the plant decreases as Na^+^ rises [6]. A major growth constraint of salt stress is a K^+^ deficiency caused by Na^+^; this effect can disrupt the cell metabolism. In fact, the phosphorus supply in our study led to a reduction in this effect of salinity by correcting the nutrient availability of the plant. Saline water significantly affected the leaf mineral content of quinoa (Table 2). Salinity increased the Na^+^ and K^+^ uptake by shoots and affected other nutrients’ balances [12,46]. Supporting our finding, i.e., the Na^+^/K^+^ ratio increased with the increment in salinity level of water irrigation; a large decrease in the K^+^/Na^+^ ratio in response to increasing salinity has been reported in quinoa by Rezzouk et al. [8]. Our finding is consistent with the results reported by Hirich et al. [13] in quinoa, who reported that increased salinity resulted in a higher Na^+^/K^+^ ratio. Conversely, Ruiz et al. [9] reported that although the Na^+^ increase was very high, the K^+^/Na^+^ ratio (ratio of 1) needs to be considered as desired. In our study, saline water irrigation significantly increased sodium (Na+) uptake, while it reduced Mg^2+^, Ca^2+^, and Fe^2+^ in quinoa leaf (Table 2). Our results are in agreement with those obtained by Maleki et al. [22] in quinoa leaves under salinity conditions. Regarding the ratios Ca^2+^/Na^+^ and Mg^2+^/Na^+^, they have been significantly reduced with increasing salinity levels. Our results support the finding of Rezzouk et al. [8] who obtained similar results in quinoa. The total carbon and the leaf dry matter were also significantly decreased under saline conditions, while nitrogen content increased.

## 4. Materials and Methods

### 4.1. Experimental Site

This research was conducted in the experimental farm on the National Institute of Agricultural Research in Foum el Oued area, Laayoune, south of Morocco (X = 27.176°; Y = −13.349°; Z = 37 m). The soil in the experimental site was sandy loam (61% sand, 18% silt, and 18% clay), moderately saline and poor in organic matter and nutrients (Table 3).

Foum El Oued area has a desert climate. The three years of climatic data (Table 4) showed an average temperature of 20 °C, a total of 18 rainy days in a year with an average annual rainfall of 72 mm, and an annual average wind speed of 24.3 km·h^−1^. In the experimental site, August is the warmest month of the year and January is the coldest month.

### 4.2. Experimental Detail and Crop Management

**Figure 5:**
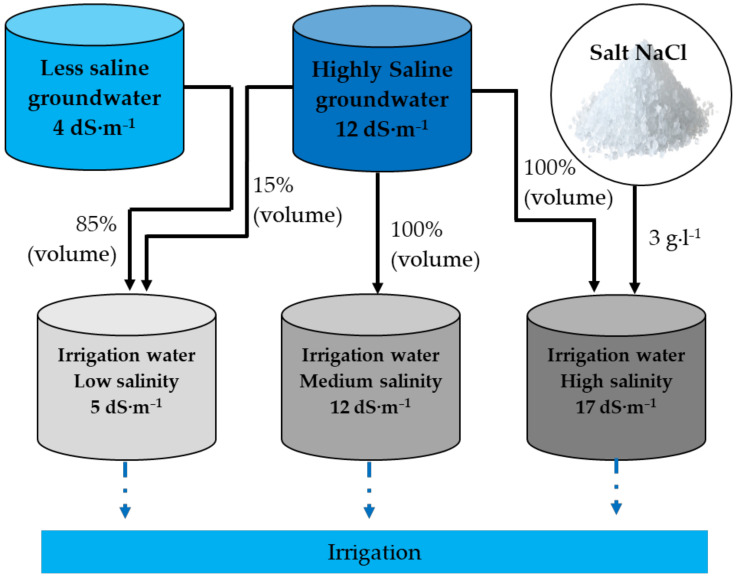
Schematic view of the irrigation water tanks laid out for using irrigation in the experimental plots.

This research was conducted between March and July 2020 at the National Institute of Agronomic Research (INRA) experimental station in Foum el Oued, Laayoune. Quinoa *Chenopodium quinoa* Willd. variety ICBA-Q5 (Origin: Coast, Chile) was used in this experiment, applying three salinity levels of irrigation water (main-plot) and three rates of phosphorus (sub-plot treatment) organized in a split-plot design with three replications. The phosphorus treatments consisted of 0, 60, and 70 kg of P_2_O_5_ ha^−1^, and the applied irrigation water salinity levels included ECs of 5, 12, and 17 dS·m^−1^. The area of each plot was 6.12 m^2^ and each consisted of five rows with 30 cm between each. The crop was sown manually on 5 March 2020. The crop spacing was kept at 30 cm row to row and 5 cm plant to plant. Irrigation was delivered using surface drip irrigation with a flow rate of 2 L·h^−1^, and the distance between drippers was equal to 33 cm. Saline irrigation water treatments were prepared using two sources of irrigation water (Table 5) in addition to salt (NaCl), as shown in Figure 5.

Three phosphorus fertilizations were applied based on plant requirements and consisted of 0% (without phosphorus input), 100, and 120% of the crop phosphorus requirements, which corresponded to 0, 60, and 70 kg of P_2_O_5_ ha^−1^, respectively. Fertilizer mono-ammonium phosphate (MAP) with 61% P_2_O_5_ was applied. The other fertilizer requirements were applied equally for all treatments. The nitrogen supply was adjusted accordingly for 0 and 60 kg of P_2_O_5_ ha^−1^ as MAP fertilizer also contains nitrogen (12% of N). The saline treatment was applied directly after sowing, and the crop was irrigated twice a week until harvest (15 July 2020) following the evapotranspiration method (ET0). The total water supply for irrigation during the growing period was equal to 250 mm (Table 6).

The harvest was carried out manually depending on the maturity of each experimental unit. We started the harvest on 10 July and the last harvest was carried out on 26 July 2020. After the harvest, the quinoa was put in bags for weighing to determine the total biomass yield, and then the plants were dried in a well-ventilated shade house. After drying, the harvest of each plot was threshed to extract the seeds.

### 4.3. Observations

#### 4.3.1. Seed and Dry Matter Yield

Seed and total dry biomass yields were measured at harvest, from all plants in the plots. The harvest index (HI) was calculated as the ratio of seed yield to total dry biomass.

#### 4.3.2. Crop and Irrigation Water Productivity

The crop water productivity (CWP) was calculated by dividing the seed yield by the total irrigation amount. Irrigation or biomass water productivity (IWP) was calculated by dividing the total biomass by the total irrigation amount.

#### 4.3.3. Chemical Analysis

Samples of fresh leaves were dried at 70 °C until they reached a constant weight before grinding into a fine powder for macro and micronutrient concentration analysis. Total nitrogen (N) was determined using the microKjeldahl method. Potassium (K^+^) and sodium (Na^+^) in leaves were determined in plant samples by a wet digestion procedure using a mixture of nitric acid and perchloric acid with a ratio of 2:1 using a flame photometer according to the method described by Chapman and Pratt [49]. P (%) was determined by colorimetry using the stannous chloride and ammonium molybdate reagent, as described by King (1951), after its extraction with sodium bicarbonate according to Olsen [50]. Calcium (Ca), Magnesium (Mg), Iron (Fe), and Zinc (Zn) were determined in plant and soil samples using methods described by Ryan et al. [51]. Soil salinity was measured using the 1:5 aqueous extract method [52,53,54]. OC was measured following the dry combustion method using an elemental analyzer.

### 4.4. Statistical Analysis

Statistical analysis was performed using SPSS 17.0 software. Two-way analysis of variance (ANOVA) was used to assess the effects of salinity and phosphorus on the monitored parameters. For each analysis, when the ANOVA was significant, statistically significant differences were identified between the means using the Tukey test (*p* ≤ 0.05). The level of significance was set to *p* < 0.05. Correlation and multivariate analysis and visualization were performed using the statistical programming language R 4.0.5. The “corrplot” package was used to investigate the strength of the linear relationship between two variables based on the Pearson correlation coefficient, and the level of significance was set to *p* < 0.05. Principal component analysis (PCA) was performed using “ggplot2,” “factoextra,” and “FactoMineR” packages.

## 5. Conclusions

This study quantified the responses of quinoa (in terms of seed and dry matter yield, crop and irrigation water productivity, and leaf mineral nutrient content) to different levels of irrigation water salinity and phosphorus fertilizer rates. Quinoa was shown to be tolerant to salinity up to 17 dS·m^−1^ without a notable yield loss. This research highlighted the great potential of improving quinoa yield through the application of the optimal amount of phosphorus fertilizer in salt-affected areas. The findings of this study revealed that phosphorus fertilization significantly increased the biomass and seed yield of quinoa under a high salinity level, which has a significant implication in sustaining the food, forage, and nutritional system in the region. It can be recommended to apply phosphorus up to a rate of 70 kg·ha^−1^ under an EC up to 17 dS·m^−1^ for good biomass harvest, while for good seed yield, an application of 70 kg·ha^−1^ phosphorus under an EC of 5 dS·m^−1^ is recommended. However, if the EC is higher than 12 dS·m^−1^, a phosphorus rate of 60 kg·ha^−1^ gives the maximum seed yield of quinoa. In light of the results obtained, we recommend conducting similar field tests on other crops and in other salt-affected regions in Morocco and Africa to confirm the findings of this study.

## Figures and Tables

**Table 1:** Analysis of variance (ANOVA) showing p-value of different leaf nutrient contents as affected by different irrigation water salinities and phosphorus rates. DF: degree of freedom, DM: dry matter, SY: seed yield, HI: harvest index, CWP: crop water productivity, IWP: irrigation/biomass water productivity, OC: organic carbon, N: Nitrogen, P: Phosphorus, K: Potassium, Mg: Magnesium, Ca: Calcium, Zn: Zinc, Fe: Iron, Na: Sodium. Values in the table are *p*-values, * denotes *p* < 0.05, ** denotes *p* < 0.01, *** denotes *p* < 0.001; ns = not significant.

Factors	DF	DM	SY	HI	CWP	IWP	Leaf Nutrient Content								
							OC	N	P	K	Mg	Ca	Zn	Fe	Na
Irrigation water salinity (S)	2	0.018 *	0.014 *	0.00 ***	0.005 **	0.013 *	0.047 *	0.023 *	0.032 *	0.021	0.044 *	0.047 *	0.54	0.26	0.010 **
Phosphorus rate (P)	2	0.027 *	0.032 *	0.047 *	0.033 *	0.02 *	0.045 *	0.43	0.021 *	0.036 *	0.029 *	0.23	0.18	0.31	0.024 *
Interaction (S × P)	4	0.90 ns	0.14 ns	0.018 *	0.148 ns	0.975 ns	0.029 *	0.37	0.039 *	0.043 *	0.170 ns	0.049 *	0.270 ns	0.63 ns	0.025 *

**Table 2:** Macro- and micro-nutrient content in quinoa leaves as affected by irrigation water salinity and phosphorus fertilizer rate. Any two values within a column are significantly different (*p* ≤ 0.05) if they have no letter in common. Small and same letters (a, ab, b) indicate the statistically homogeneous groups within phosphorus fertilization treatments, and capital and same letters (A, AB, B) indicate the statistically homogeneous groups within salinity treatments.

Irrigation Water EC	P Rate (kg of P_2_O_5_·ha^−1^)	OC (%)	N (%)	P (%)	K (%)	Mg (%)	Ca (%)	Zn (ppm)	Fe (ppm)	Na (%)	K/Na	Ca/Na	Mg/Na
5 dS·m^−1^	0	40.4 ± 8.4 a	1.4 ± 0.1 a	0.14 ± 0.03 a	5.3 ± 1 b	2.3 ± 0.2 a	3.1 ± 1.1 a	22.6 ± 3 b	343 ± 29 b	3.3 ± 0.6 b	1.6 ± 0.2 a	1.1 ± 0.6 a	0.7 ± 0.2 a
	60	40.0 ± 6.3 a	1.2 ± 0.1 a	0.12 ± 0.03 b	6.4 ± 2 a	2.2 ± 0.3 a	2.6 ± 1 b	26.1 ± 6 a	324 ± 17 b	3.5 ± 0.2 a	1.8 ± 0.4 a	0.8 ± 0.4 b	0.6 ± 0.1 b
	70	36.2 ± 2.3 a	1.4 ± 0.1 a	0.15 ± 0.02 a	5.7 ± 1 ab	2.2 ± 0.1 a	2.8 ± 0.6 ab	22.1 ± 3 b	433 ± 16 a	3.6 ± 0.3 a	1.6 ± 0.6 a	0.8 ± 0.1 b	0.6 ± 0 b
	**Average**	**38.9 ± 5.7 A**	**1.3 ± 0.1 B**	**0.14 ± 0.02 B**	**6 ± 1.5 B**	**2.2 ± 0.2 A**	**3 ± 0.9 A**	**24 ± 4 A**	**366 ± 69 A**	**3 ± 0.4 B**	**2 ± 0.4 A**	**0.9 ± 0.4 A**	**0.7 ± 0.1 A**
12 dS·m^−1^	0	35.1 ± 1.8 a	1.5 ± 0.1 a	0.30 ± 0.13 b	7.6 ± 1 b	2 ± 0.1 b	1.7 ± 0.1 b	28.8 ± 7 ab	280 ± 13 a	4.6 ± 1 a	1.7 ± 0.3 b	0.4 ± 0.1 c	0.5 ± 0.1 b
	60	34.3 ± 0.7 b	1.7 ± 0.2 a	0.34 ± 0.07 b	8.6 ± 1 a	2 ± 0.3 b	1.9 ± 0.3 ab	24.1 ± 5 b	251 ± 16 a	4.3 ± 0.3 ab	2 ± 0.4 a	0.4 ± 0 b	0.5 ± 0 b
	70	35.2 ± 1.7 a	1.6 ± 0.1 a	0.41 ± 0.24 a	8.1 ± 0 ab	2.4 ± 0.2 a	2.1 ± 0.2 a	33.3 ± 17 a	212 ± 6 a	4 ± 0.1 b	2 ± 0.1 a	0.5 ± 0.1 a	0.6 ± 0.1 a
	**Average**	**34.8 ± 1.4 AB**	**1.6 ± 0.1 A**	**0.35 ± 0.15 A**	**8 ± 0.7 A**	**2.1 ± 0.2 AB**	**2 ± 0.2 B**	**29 ± 10 A**	**248 ± 57 A**	**4 ± 0.5 A**	**2 ± 0.3 A**	**0.5 ± 0.1 B**	**0.5 ± 0.1 B**
17 dS·m^−1^	0	34.8 ± 1.1 a	1.6 ± 0.1 a	0.25 ± 0.03 a	8.2 ± 1 c	1.9 ± 0.1 b	1.6 ± 0.1 b	15.3 ± 2 b	248 ± 15 a	4.5 ± 0.3 b	1.8 ± 0.1 ab	0.4 ± 0 b	0.4 ± 0 ab
	60	34.3 ± 1.3 a	1.6 ± 0 a	0.26 ± 0.06 a	9.3 ± 0 a	2.1 ± 0.2 a	1.7 ± 0.2 a	27.6 ± 3 a	244 ± 15 a	4.5 ± 0.3 b	2.1 ± 0.2 a	0.4 ± 0 a	0.5 ± 0 a
	70	31.7 ± 1.7 b	1.5 ± 0.1 a	0.23 ± 0.04 b	9.1 ± 0 b	1.9 ± 0.2 b	1.6 ± 0.2 b	22.7 ± 8 ab	214 ± 12 a	5.5 ± 0.6 a	1.7 ± 0.2 b	0.3 ± 0 b	0.3 ± 0 b
	**Average**	**33.6 ± 1.8 B**	**1.6 ± 0.1 A**	**0.25 ± 0.04 AB**	**9 ± 0.3 A**	**1.9 ± 0.2 A**	**2 ± 0.1 A**	**22 ± 3 A**	**235 ± 34 A**	**5 ± 0.4 A**	**2 ± 0.2 A**	**0.3 ± 0.03 B**	**0.4 ± 0 B**

**Table 3:** Initial soil physical and chemical properties in the experimental site.

Depth (cm)	Sand (%)	Silt (%)	Clay (%)	Soil pH	EC1:5 (dS·m^−1^)	Cl (g·kg^−1^)	Na_2_O (g·kg^−1^)	OM (%)	N (%)	P_2_O5 (mg·kg^−1^)	K_2_O (g·kg^−1^)	MgO (g·kg^−1^)	CaO (g·kg^−1^)	Zn (mg·kg^−1^)	Fe (mg·kg^−1^)
0–20	61.8	18.6	18.6	8.47	1.91	2.12	2.00	0.47	0.03	44.12	0.33	0.92	9.73	0.80	1.23
20–40	71.3	12.9	23.8	8.47	1.80	1.43	1.51	0.40	0.03	36.29	0.31	0.85	9.46	0.80	1.23

**Table 4:** Temperature, rainfall, relative humidity, and sunshine hours during the crop growth period in 2020 and 2016–2019 three years average (source: data recorded in L’Institut National de la Recherche Agronomique (INRA), Foum El Oued, Laayoune, Morocco).

Climatic Parameters	During the Experimental Period					3 Years Average				
	March	April	May	June	July	March	April	May	June	July
Temperature (°C)	18.3	19	20.1	21.6	23	23.8	24	25.4	26.4	28.7
Rainfall (mm)	7	2	1	0	0	1	1	0	1	0
Humidity (%)	59	62	64	68	69	Data not available				
Sunshine hours (hour. day^−1^)	8.9	9	9.3	9.6	9.7	Data not available				

**Table 5:** Chemical properties of irrigation water applied.

Water Content	EC	pH	Cations (meq·L^−1^)				Anions (meq·L^−1^)				
	(dS·m^−1^)		K^+^	Na^+^	Ca^2+^	Mg^2+^	Cl^−^	SO_4_^2−^	NO_3_^−^	CO_3_^2−^	HCO_3_^−^
Freshwater	4.04	7.45	0.883	24.35	11.25	6.48	28.12	11.21	3.46	0.0	3.52
Groundwater	11.98	7.35	3.44	114.07	28.4	26.42	124.55	52.15	1.01	0.0	3.88

**Table 6:** Total irrigation water (mm) applied in each irrigation during the crop growing period from March to July.

Periods (10 Day)	March	April	May	June	July
0–10 days	20	18	27	27	9
11–20 days	-- (rain)	18	27	18	
21–31 days	18	27	27	9	
**Total irrigation (mm/month)**	**38**	**63**	**81**	**54**	**9**

## Data Availability

Not applicable.
